# Experimental study on the significance of pressure relief effect and crack extension law under uniaxial compression of rock-like materials containing drill holes

**DOI:** 10.1038/s41598-024-51490-0

**Published:** 2024-01-11

**Authors:** Lianhai Tai, Chong Li, Yin Hu, Xiaoxiao Yu, Zhijun Xu, Xiaowu Zhang, Shiguang Chai, Peng Zhang, Shihui Lu

**Affiliations:** 1 School of Mines, China University of Mining and Technology, Xuzhou, 221116 China; 2 MOE Key Laboratory of Deep Coal Resource Mining, China University of Mining and Technology, Xuzhou, 221116 China; 3 Powerchina HuaDong Engineering Corporation Limited, Hangzhou, 311122 China

**Keywords:** Mechanical properties, Civil engineering

## Abstract

The drilling pressure relief technology is an effective way to reduce the accumulation of elastic energy in the tunnel envelope, which can reduce the risk of regional ground pressure occurrence. However, there is a lack of theoretical guidance on which drilling parameter has the greatest degree of influence on the effectiveness of pressure relief. The uniaxial compression tests were conducted to study the relationships between drilling parameters (the diameter, depth, and spacing) and the mechanical properties and deformation modulus of specimens. The results show that: (1) The drilling diameter (DDR) and drilling depth (DDH) of single-hole specimens negatively correlate with the peak-failure strength and deformation modulus, while the drilling spacing (DS) of double-hole specimens positively correlates with the peak-failure strength and deformation modulus. It shows that the borehole diameter has a more significant effect on the decompression effect. (2) With the help of the Grey Relational Analysis, the factors affecting the peak-failure strength and deformation modulus of the drilled specimens were ranked in significance. From the largest to the smallest, they are DDR, followed by DDH and DS. (3) The role of the pressure relief mechanism is to transfer the high stress in the shallow part of the roadway to the deep part, reduce the peak strength of destruction and deformation modulus of the peripheral rock in the drilled section, so that the characteristics of the mechanical behavior of the rock are significantly weakened, and the range of the area of the drilled hole decompression is enlarged. During the loading of the borehole, the borehole stress field dominates in the early stage, and cracking starts near the borehole along the direction perpendicular to the direction of maximum principal stress (horizontal direction). In the later stage, the maximum principal stress field dominates and vertical cracks with large widths appear. During crack expansion, the plastic energy dissipation effect is enhanced and the deep impact conduction path is weakened, thus protecting the roadway. This study determined the significance of the pressure relief effect of different drilling parameters, which can guide reasonable modifications of drilling parameters in the field.

## Introduction

Coal is a vital energy source globally, and many problems still exist to be solved during production. With a long period of high-intensity mining, the rapid reduction of shallow and central resource reserves led to many mining areas entering the deep mining stage^1–3^. In addition, due to various factors such as abnormal geological areas, mining methods, and coal mining technology, the stressful environment of the working face changes significantly. Excessive concentration of stress easily causes dynamic pressure in roadways^4,5^, resulting in a series of problems such as fast and excessive deformation in the roadway surrounding rock and failure to maintain stability^6–10^.

For general underground engineering, strengthening the support can ensure the stability of the surrounding rock structure to a certain extent^11–13^. However, for large deformation and failure caused by dynamic pressure, if ordinary support methods are adopted, the effect often cannot meet underground safe production requirements, resulting in substantial support costs. Changing the surrounding rock stress field distribution and optimizing the surrounding rock stress environment using pressure relief is necessary^14–16^. Currently, the most critical local pressure relief techniques for dynamic pressure roadways include blasting, roof cutting, slotting, and drilling. The basic principle for these is to transfer high stress to deep rock strata, ensure that the surrounding rock support system is in a low-stress environment, and reduce roadway deformation^17,18^. He^19^ proposed a coupled method of decompression and support, which ensured the safety and stability of surrounding rock by blasting pressure relief combined with anchor cable support. Yang^20^ cut off the stress propagation path by pre-cracking and blasting the hard rock layer of the roadway, improving the stress environment of the roadway surrounding rock and controlling roadway deformation. Ma^21^ used numerical simulation to establish a three-dimensional numerical test model of single-hole uncoupled charge to determine the energy evolution and crack propagation in the blasting rock mass under the uncoupled charge structure. In addition, the top-cutting and pressure relief technique^22–26^, based on the key layer theory and the coal pillar-free retention technique, can reduce the stress in the surrounding rock of the roadway. Finally, water jets and rotary cutting tools can groove the coal seam and release the surrounding rock stress^27,28^. However, the above means of pressure relief have defects, blasting technology if improperly operated is prone to induce impact, and the coal body after blasting has a dynamic expansion effect, which is prone to cause safety accidents. Hydraulic fracturing roof-cutting technology to a certain extent destroys the integrity and stability of the roadway roof and has potential safety implications for the roadway envelope support and single-track crane transportation at the working face. With the advantages of small disturbance, convenient construction, strong applicability, and significant effect, drilling pressure relief has become the prevention and control method adopted by most impact mines^29,30^.

Scholars have conducted numerous studies on stress distribution, displacement evolution law, drilling pressure relief parameter optimization, and drilling pressure relief effect evaluation of drilling coal-rock mass^31–34^. For example, Zhai^30^ used the triaxial loading experimental system to simulate the lateral stress of coal-rock mass and used acoustic emission (AE) to monitor the AE events in different drilling processes and record their characteristics. Zhang^35^ studied the generation and development of local fractures around drilling holes and believed that the greater the density of drilling holes, the more developed the fractures and the more energy released, creating a better pressure relief effect. Wu^29^ studied the influence of hole shape on mechanical properties and fracture characteristics of hole-bearing rock under uniaxial load and analyzed the crack propagation, expansion, and stress distribution of different types of samples. They found circular holes had the best stability, followed by inverted U-shaped, trapezoidal, square, and finally rectangular. Zhao^36^ used a physical model and AE technology to study the fracture evolution of preformed circular hole rock. They learned that tensile splitting cracks were generated parallel to the loading direction, and compression cracks were generated on both sides of the hole. Lin^32^ studied the crack initiation, coalition mechanism, and failure behavior of preformed pore granite samples with different pore sizes, distribution, and spacing types. Zhao^34^ studied the influence of borehole arrangement on the mechanical properties of the coal model through a uniaxial compression test. They determined the internal relationship between borehole diameter, borehole row number, and energy evolution (energy dissipation and blasting energy index). Yao^37^ simulated the mechanism of large-diameter drilling pressure relief technology and its influence on anchorage support. They put forward the segmental large-diameter drilling pressure relief technology.

Overall, the above research has studied surface deformation and failure mechanisms of different prefabricated coal-rock samples based on fracture development. However, these studies were from the perspective of stress. Drilling parameters will not only affect the deformation characteristics and stress evolution of coal and rock masses but also lead to apparent differences in the mechanical properties and pressure relief effects of coal and rock mass if the borehole size is different. There is a gap in the experimental research on the significance of the size effect and pressure relief effect of pressure relief drill holes.

Although Cui^38^ proved through simulation research that bore diameter was the main controlling factor affecting the pressure relief effect, they did not effectively prove it with the help of experimental research. The significance of the size effect and pressure relief effect of the hole-containing coal rock specimens is critical and can provide a practical guide to ensure the safe production of impact-hazardous working faces. The above research results have a vital role in promoting the development of pressure relief technology in the borehole of the working face dynamic pressure roadway; however, there are essential differences between the geological conditions and the actual situation in each mining area. For mines with more complicated production conditions and particularly prominent historical legacy problems, there is a lack of practical guidance on the issue of improving the pressure relief effect when only a particular parameter of the borehole pressure relief can be changed. For example, when the size of the section coal pillar is small, the depth of the borehole should not be too large, which would be better for pressure relief. Therefore, the question is whether to adjust the borehole diameter or the spacing. The Grey Relational Analysis can express the degree of correlation between different factors and make predictions and decisions accordingly and is widely used in engineering, economics, management, and other fields^39–41^. In summary, through Grey Relational Analysis, it can be seen that the study on the significance of the pressure relief effect of different drilling parameters is of great significance for guiding production underground.

In this paper, through indoor experimental studies, the link between the effect of borehole size and the strength, deformation modulus, and fracture development characteristics of coal rock specimens was determined. The significance of the pressure relief effect of different borehole parameters was also determined with the help of Grey Relational Analysis. The research results of this paper can guide the adjustment of borehole parameters in mines, and the correct modification of borehole parameters can realize the rapid and efficient release of energy from the working face under restricted production conditions. This will help to reduce the rock burst of the working face.

## Geological background and drilling pressure relief principle

### The engineering status

**Figure 1:**
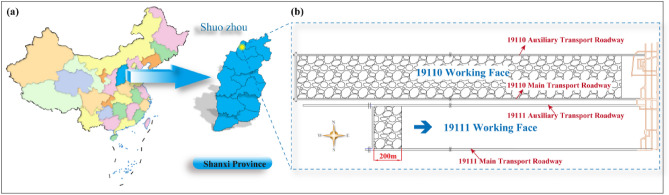
Engineering geological map of Pingshuo No.1 Coal Mine: (**a**) Geographic location; (**b**) Layout of 19,111 working face.

The Pingshuo No.1 Coal Mine is located in Pinglu District, Shuozhou City, Shanxi Province, with a verified production capacity of 10 Mt/a. The main coal seam is Coal Seam 9 #, with an average dip of 3.5° and an average coal thickness of 11.3 m. Currently, the 19,111 working face is being mined, with 200 m already mined. The engineering geological map is shown in Fig. 1.

### Drilling pressure relief principle

The radius of a single borehole pressure relief zone is mainly affected by vertical stress, lateral pressure coefficient, surrounding rock cohesion, internal friction angle, and borehole radius^42^. Only borehole parameters can be manually changed for underground mining activities, so the influence of borehole parameters on specimen strength, deformation modulus, and failure characteristics can be studied through laboratory tests.

**Figure 2:**
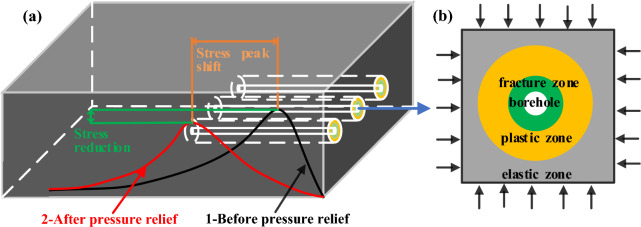
Drill hole pressure relief principle: (**a**) Stress curve distribution; (**b**) Surrounding rock zoning.

The excavation of the roadway broke the equilibrium state of the original rock stress field^43^, and after the stress redistribution, the surrounding rock formed a fracture zone, plastic zone, and elastic stress elevation zone (elastic zone), as shown in Fig. 2b from shallow to deep. The junction of the plastic zone and the elastic stress elevation zone is the location of the peak stress, as shown in curve 1 in Fig. 2a. After constructing multiple boreholes inside the roadway, the borehole surrounding rock also formed a fracture zone, a plastic zone, and an elastic stress elevation zone from shallow to deep due to the redistributed stress. When multiple borehole fracture zones and plastic zones interact, a large pressure relief circle forms in the roadway's pressure relief section. The reduction of the bearing capacity of the surrounding rock led to the transfer of high stress from the shallow part to the deep part, and the stress at the original peak position was reduced, as shown in curve 2 in Fig. 2a, to achieve pressure relief.

## Materials and methods

### Materials

Sand, cement, and gypsum were selected as similar materials for proportioning. The bulk density was γ = 18 kN/m^3^. The aggregate was naturally graded ordinary river sand, and the natural gradation was as listed in Table 1. The cement was white silicate cement grade 325. Gypsum was a common gypsum powder. The 9 # coal mechanical parameters are shown in Table 2. The similarity ratio of capacity to weight was calculated as *C*_*γ*_ = 12.3/18 = 0.68. The similarity ratio of stress was calculated as *C*_*σ*_ = *C*_*l*_ × *C*_*γ*_ = 10 × 0.68 = 6.8, and the uniaxial compressive strength of the test model design was *C*_*c*_ = *C*_*coal*_ × *C*_*σ*_ = 35.79/6.8 = 5.26 MPa. Multiple groups of similar material proportioning tests were conducted to determine the mass ratio of cement: gypsum: water: sand as 1:3:3.5:10.

**Table 1:** Natural gradation of sand.

Diameter of sand (mm)	> 1.18	0.6–1.18	0.3–0.6	0.15–0.3	0.075 –0.15	< 0.075
Percentage(%)	0.361	0.794	1.733	64.621	28.520	3.971

**Table 2:** Mechanical parameters of coal and similar models.

Material	Bulk density(kN/m^3^)	Uniaxial compressive strength(MPa)	Poisson's ratio
Coal	12.3	35.79	0.31
Similar model	18	5.26	0.26

### Methods

**Figure 3:**
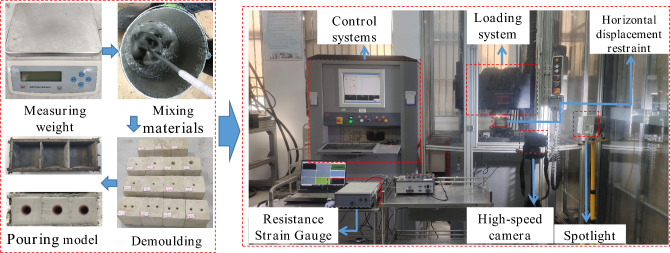
The experimental process and equipment.

Figure 3 shows the experimental process and equipment. The specific steps are given as follows:

- The geometric similarity ratio of the model test was taken as follows. The length, width, and height of the model were 100 mm × 100 mm × 100 mm. The holes were reserved inside the model, and the horizontal displacement of the fixed specimen was fixed during the test.
- The control variable method was used. Combined with the site construction situation, the three parameter sizes differ by 1 ~ 2 orders of magnitude, so the similarity ratios cannot be consistent. For the special case of drilling depth of 6 ~ 24 m (coal pillar side), the commonly used drilling spacing is 1.5 ~ 2 m, and the drilling diameter is 100 ~ 300 mm, so the similarity ratios are determined to be 10 for the drilling diameter (DDR), 300 for the drilling depth (DDH), and 50 for the drilling spacing (DS). Three specimens of the same size were tested in each group. The specific research plan is given as follows. (1) The complete specimens without boreholes are named C1, C2, C3. (2) To study the effect of borehole diameter, the specimen contained only a single hole with a fixed borehole depth of 100 mm and borehole diameters of 10 mm, 15 mm, 20 mm, or 25 mm. Name them in order as R1-1, R1-2, R1-3…R4-2, R4-3. (3) To study the effect of borehole depth, the specimen contained only a single hole with a fixed borehole diameter of 10 mm and borehole depths of 20 mm, 40 mm, 60 mm, or 80 mm. Name them in order as H1-1, H1-2, H1-3 … H4-2, H4-3. (4) To study the effect of borehole spacing, the specimens were tested with double holes, with a fixed borehole diameter of 10 mm, a borehole depth of 100 mm and borehole spacing of 30 mm, 35 mm, 40 mm, or 45 mm. Name them in order as S1-1, S1-2, S1-3 … S4-2, S4-3. A specimen was taken from each group to make a test protocol, and the details are shown in Table 3.
- A layer of cling film was wrapped around the outer wall of the PVC pipe, which had different diameters. It was fixed inside the mold according to the experimental design scheme, after which it was poured. The specimens were demolded 48 h after production, and the PVC pipe with cling film was removed with tools and placed in the laboratory for support.
- The test loading unit adopted C64.106/1000 kN electro-hydraulic servo universal testing machine, and the control unit adopted Test Expert. NET control software with a sampling frequency of 20 Hz. The test model horizontal displacement restraint device consists of two high-strength horizontal restraint steel plates, four M10 restraint steel rods, and M10 hexagonal nuts. The test was loaded at a rate of 0.8 mm/min until the post-peak strength of the specimen model was 70%. The test process was filmed with a high-speed camera at a recording speed of 50 frames/s.

**Table 3:**
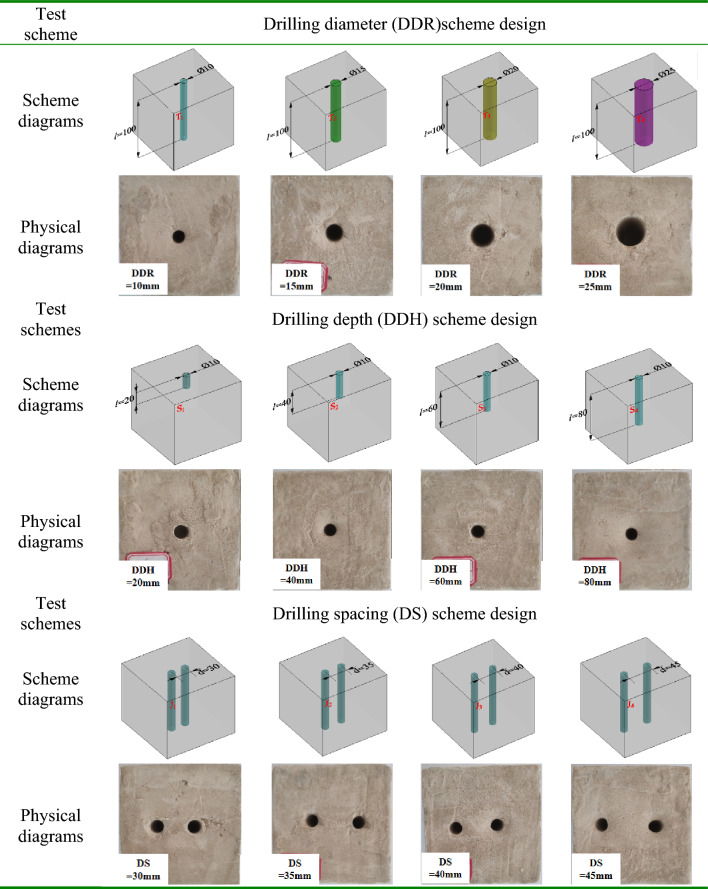
Scheme design of different drilling diameters (unit: mm).

## Results and discussion

### Effects of drilling parameters on the specimens failure strength and deformation modulus

The absolute deviation (the absolute deviation of each test result from the mean value), relative deviation (the ratio of the absolute deviation to the mean value), standard deviation and relative standard deviation of the mean values for each drilling parameters are also provided in Tables 4, 5, 6, 7, 8, 9 to examine the reproducibility of the test results. The above data were also plotted in Figs. 7, 10, 13. Both the relative deviation and relative standard deviation in Tables 4, 5, 6, 7, 8, 9 are lower than 15% with the maximum relative deviation and the maximum coefficient of deviation being 13.98% (Specimen number H_1-2_ in Table 7) and 9.92% (Test Scenario DDR = 25 mm in Table 5) respectively. The deviation values are within acceptable limits and prove the reproducibility of the test results^44^. The values in each test scenario in Tables 4, 5, 6, 7, 8, 9 are used in the following sections to study the effect of drilling parameters on specimen peak strength and specimen deformation modulus.

**Table 4:** Experimental results of the peak-failure strength with different DDRs.

Specimen number	Strength (MPa)	Absolute deviation (MPa)	Relative deviation (%)	Mean value (MPa)	Standard deviation (MPa)	Coefficientof variation (%)
C1	5.75	0.02	0.41	5.73	0.14	2.51
C2	5.89	0.16	2.81			
C3	5.54	0.19	3.31			
R_1-1_	4.18	0.58	12.91	4.76	0.46	9.70
R_1-2_	5.31	0.55	10.99			
R_1-3_	4.78	0.02	0.49			
R_2-1_	4.62	0.34	7.56	4.28	0.38	8.90
R_2-2_	3.75	0.53	13.28			
R_2-3_	4.48	0.20	4.49			
R_3-1_	3.71	0.01	0.27	3.72	0.16	4.28
R_3-2_	3.53	0.19	5.24			
R_3-3_	3.92	0.20	5.24			
R_4-1_	3.28	0.02	0.61	3.26	0.29	9.03
R_4-2_	2.89	0.37	12.03			
R_4-3_	3.61	0.35	10.19

**Table 5:** Experimental results of the deformation modulus with different DDRs.

Specimen number	Deformation modulus (MPa)	Absolute deviation (MPa)	Relative deviation (%)	Mean value (MPa)	Standard deviation (MPa)	Coefficient of variation (%)
C1	0.75	0.12	14.81	0.87	0.07	8.02
C2	0.88	0.01	1.14			
C3	0.98	0.11	11.89			
R_1-1_	0.75	0.08	10.13	0.83	0.06	7.43
R_1-2_	0.84	0.01	1.20			
R_1-3_	0.90	0.07	8.09			
R_2-1_	0.68	0.07	10.23	0.75	0.07	9.47
R_2-2_	0.85	0.10	12.06			
R_2-3_	0.73	0.02	3.15			
R_3-1_	0.71	0.08	11.94	0.63	0.06	9.35
R_3-2_	0.61	0.02	3.23			
R_3-3_	0.57	0.06	10.00			
R_4-1_	0.61	0.07	12.17	0.54	0.05	9.92
R_4-2_	0.53	0.01	1.87			
R_4-3_	0.48	0.06	11.76

**Table 6:** Experimental results of the peak-failure strength with different DDHs.

Specimen number	Strength (MPa)	Absolute deviation (MPa)	Relative deviation (%)	Mean Value (MPa)	Standard deviation (MPa)	Coefficient of variation (%)
H_1-1_	5.25	0.37	6.75	5.62	0.26	4.63
H_1-2_	5.78	0.16	2.87			
H_1-3_	5.82	0.20	3.56			
H_2-1_	5.76	0.28	4.98	5.48	0.39	7.10
H_2-2_	4.93	0.55	10.57			
H_2-3_	5.75	0.27	4.81			
H_3-1_	4.85	0.27	5.35	5.12	0.30	5.79
H_3-2_	4.97	0.15	2.91			
H_3-3_	5.53	0.41	7.76			
H_4-1_	4.79	0.16	3.29	4.95	0.12	2.43
H_4-2_	5.08	0.13	2.59			
H_4-3_	4.98	0.03	0.60

**Table 7:** Experimental results of the deformation modulus with different DDHs.

Specimen number	Deformation modulus (MPa)	Absolute deviation (MPa)	Relative deviation (%)	Mean value (MPa)	Standard deviation (MPa)	Coefficient of variation (%)
H_1-1_	0.82	0.02	2.41	0.84	0.06	7.01
H_1-2_	0.92	0.08	9.09			
H_1-3_	0.78	0.06	7.41			
H_2-1_	0.74	0.03	3.67	0.71	0.05	7.36
H_2-2_	0.76	0.05	6.33			
H_2-3_	0.64	0.07	10.84			
H_3-1_	0.71	0.04	5.80	0.67	0.04	6.45
H_3-2_	0.69	0.02	2.94			
H_3-3_	0.61	0.06	9.37			
H_4-1_	0.51	0.08	13.98	0.59	0.06	9.87
H_4-2_	0.65	0.06	10.24			
H_4-3_	0.60	0.01	2.25

**Table 8:** Experimental results of the peak-failure strength with different DSs.

Specimen number	Strength (MPa)	Absolute deviation (MPa)	Relative deviation (%)	Mean value (MPa)	Standard deviation (MPa)	Coefficient of variation (%)
S_1-1_	4.02	0.15	3.80	3.87	0.12	2.98
S_1-2_	3.85	0.02	0.52			
S_1-3_	3.74	0.13	3.42			
S_2-1_	4.22	0.08	1.91	4.14	0.06	1.42
S_2-2_	4.12	0.02	0.48			
S_2-3_	4.08	0.06	1.46			
S_3-1_	4.56	0.35	7.90	4.21	0.36	8.50
S_3-2_	4.36	0.15	3.42			
S_3-3_	3.72	0.49	12.44			
S_4-1_	4.32	0.26	5.84	4.58	0.22	4.73
S_4-2_	4.85	0.27	5.73			
S_4-3_	4.57	0.01	0.22

**Table 9:** Experimental results of the deformation modulus with different DSs.

Specimen number	Deformation modulus (MPa)	Absolute Deviation (MPa)	Relative Deviation (%)	Mean Value (MPa)	Standard Deviation (MPa)	Coefficient of Variation (%)
S_1-1_	0.44	0.01	2.25	0.45	0.04	8.31
S_1-2_	0.50	0.05	10.53			
S_1-3_	0.41	0.04	9.30			
S_2-1_	0.46	0.01	1.44	0.47	0.04	8.81
S_2-2_	0.42	0.05	10.53			
S_2-3_	0.52	0.05	10.81			
S_3-1_	0.65	0.06	10.24	0.59	0.04	7.67
S_3-2_	0.56	0.03	4.65			
S_3-3_	0.55	0.04	6.45			
S_4-1_	0.76	0.03	4.03	0.73	0.06	8.88
S_4-2_	0.79	0.06	7.89			
S_4-3_	0.64	0.09	13.14			

#### DDR

**Figure 4:**
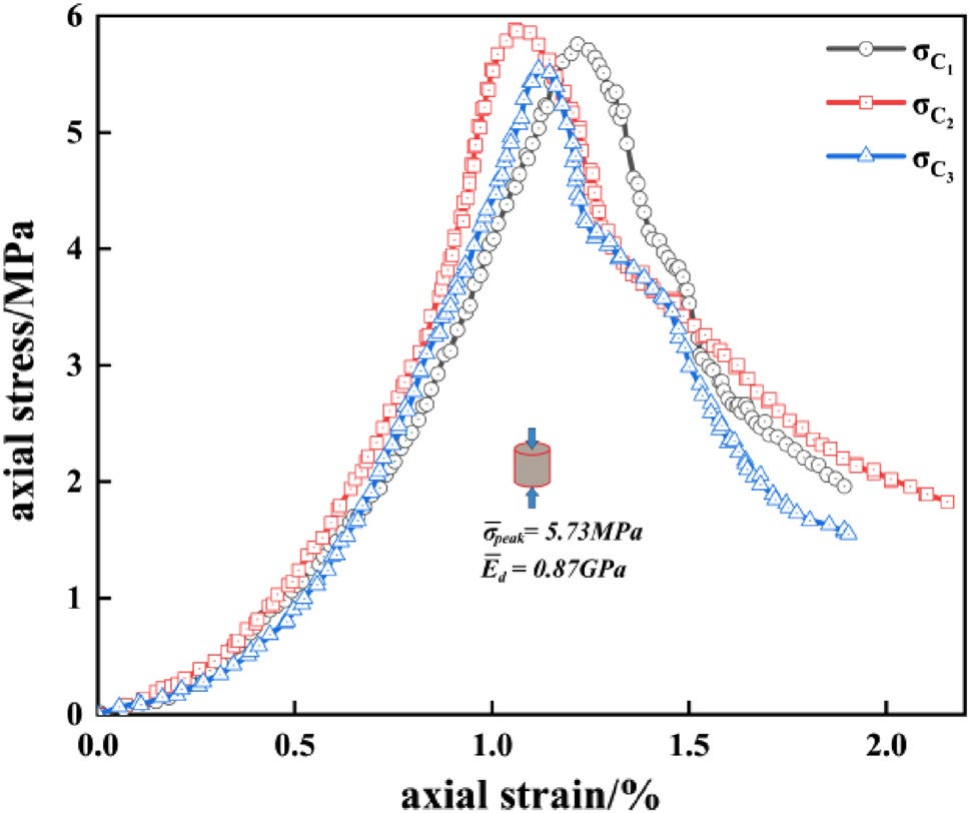
Stress–strain curves of the intact specimens.

**Figure 5:**
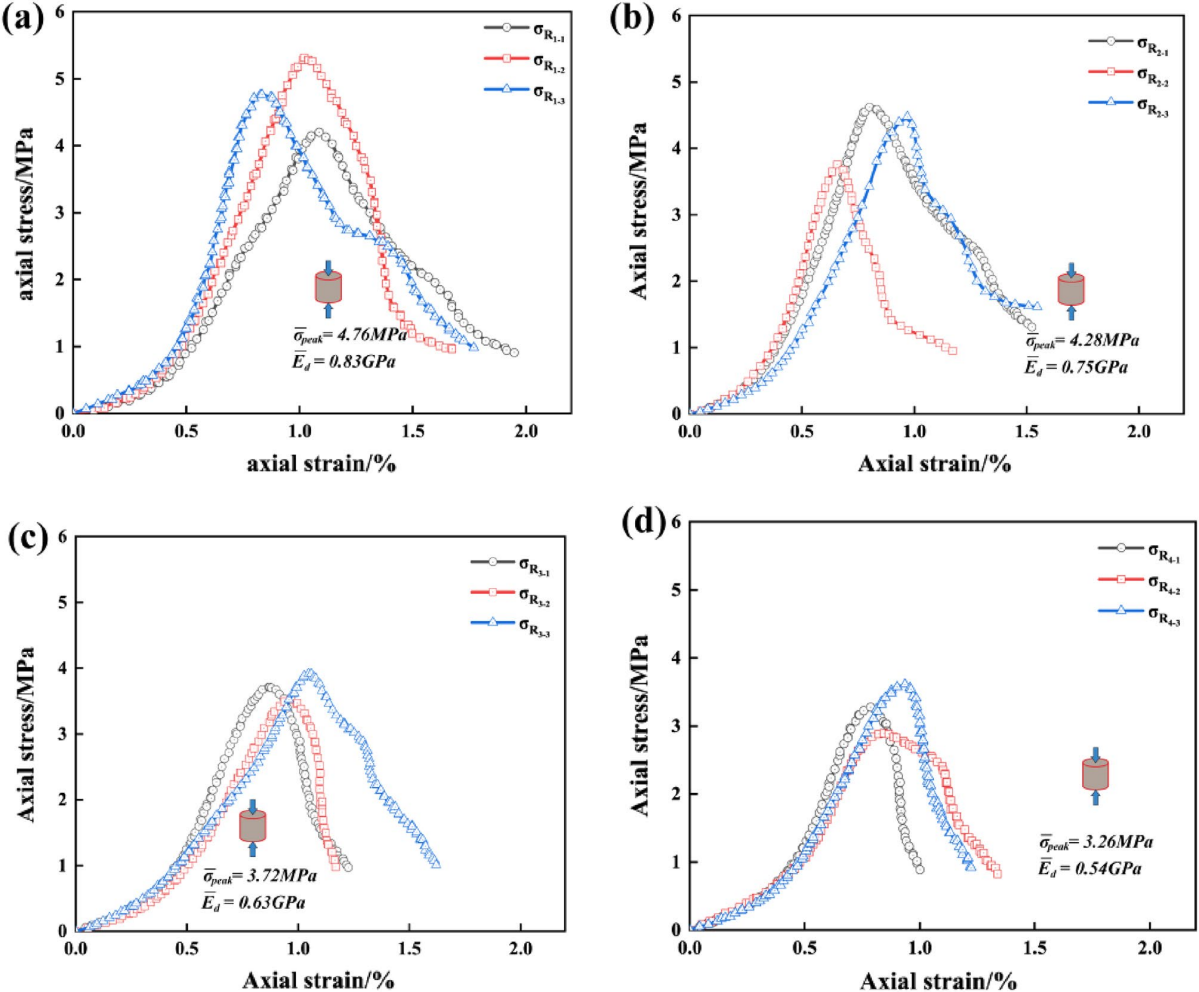
Stress–strain curves of the drilled specimens with different DDRs: (**a**) DDR = 10 mm; (**b**) DDR = 15 mm; (**c**) DDR = 20 mm; (**d**) DDR = 25 mm.

**Figure 6:**
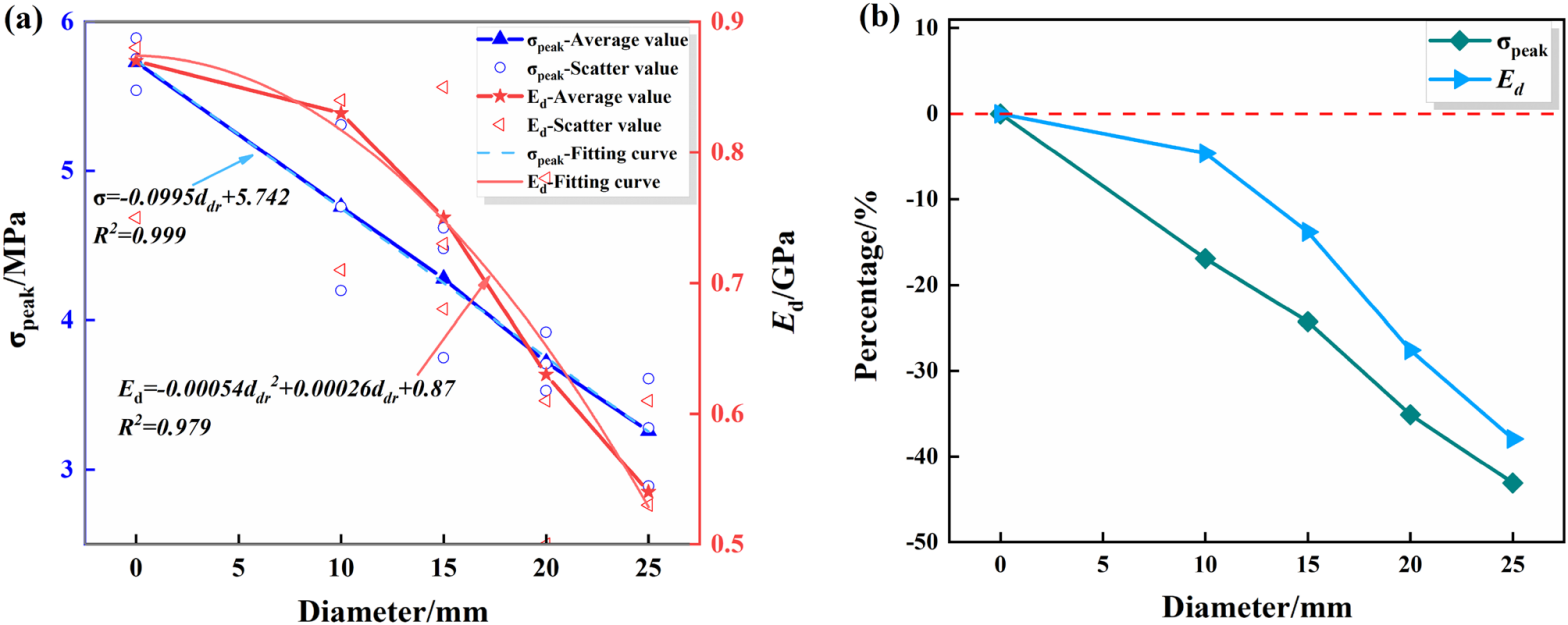
Variations of the peak strength and deformation modulus of the borehole specimens with DDRs: (**a**) Data and fitting curve; (**b**) Mean change rate.

1. Tables 4 and 5 indicate the mean value of the peak-failure strength of the intact specimen is 5.73 MPa, and the mean value of the deformation modulus is 0.87 GPa. The mean values of the peak-failure strength of the drilled specimens are 4.76 MPa, 4.28 MPa, 3.72 MPa, 3.26 MPa, and the mean values of the deformation modulus are 0.83 GPa, 0.75 GPa, 0.63 GPa, 0.54 GPa, when the borehole diameters are 10 mm, 15 mm, 20 mm, and 25 mm, respectively. The presence of the boreholes changed the stress structure of the specimens and caused the strength to decrease continuously. In addition, from Figs. 4, 5, 6, the mean values of the peak-failure strength of the four different borehole diameters decrease by 16.9%, 24.3%, 35.1%, and 43.1%, the mean values of the deformation modulus of the four different borehole diameters decrease by 4.60%, 13.79%, 27.59%, and 37.93%, respectively, compared with those of the intact specimens.
2. With the increasing drilling diameter, the peak-failure strength and the deformation modulus of the specimen decreases, and overall both of them are negatively correlated with the drilling diameter. The fitting functions are σ = − 0.0995d_dr_ + 5.742, and E = − 0.00054d_dr_^2^ − 0.00026d_dr_ + 0.87. The values of the goodness of fit are 0.999 and 0.979, respectively, and the goodness of fit is fine.
3. When the diameter of the borehole exceeds 15 mm, the stress–strain curves of large diameter specimens are more likely to enter the yielding stage during loading, and the weakening effect of strength is more obvious. this was also confirmed by Huang^45^ investigated the acoustic emission (AE) characteristics of specimens at different borehole diameters and found that the AE activity before the peak strength point was stronger when the borehole diameter was larger, resulting in the bearing capacity of the specimens is also significantly weaker. This further indicates that larger diameter boreholes produce more damage to the interior of the rock mass, resulting in a significant weakening of the rock mechanical behavior characteristics. It is also demonstrated that the larger diameter borehole creates a wider range of pressure relief area, which can better achieve stress transfer and reduce the impact hazard^46–48^.

**Figure 7:**
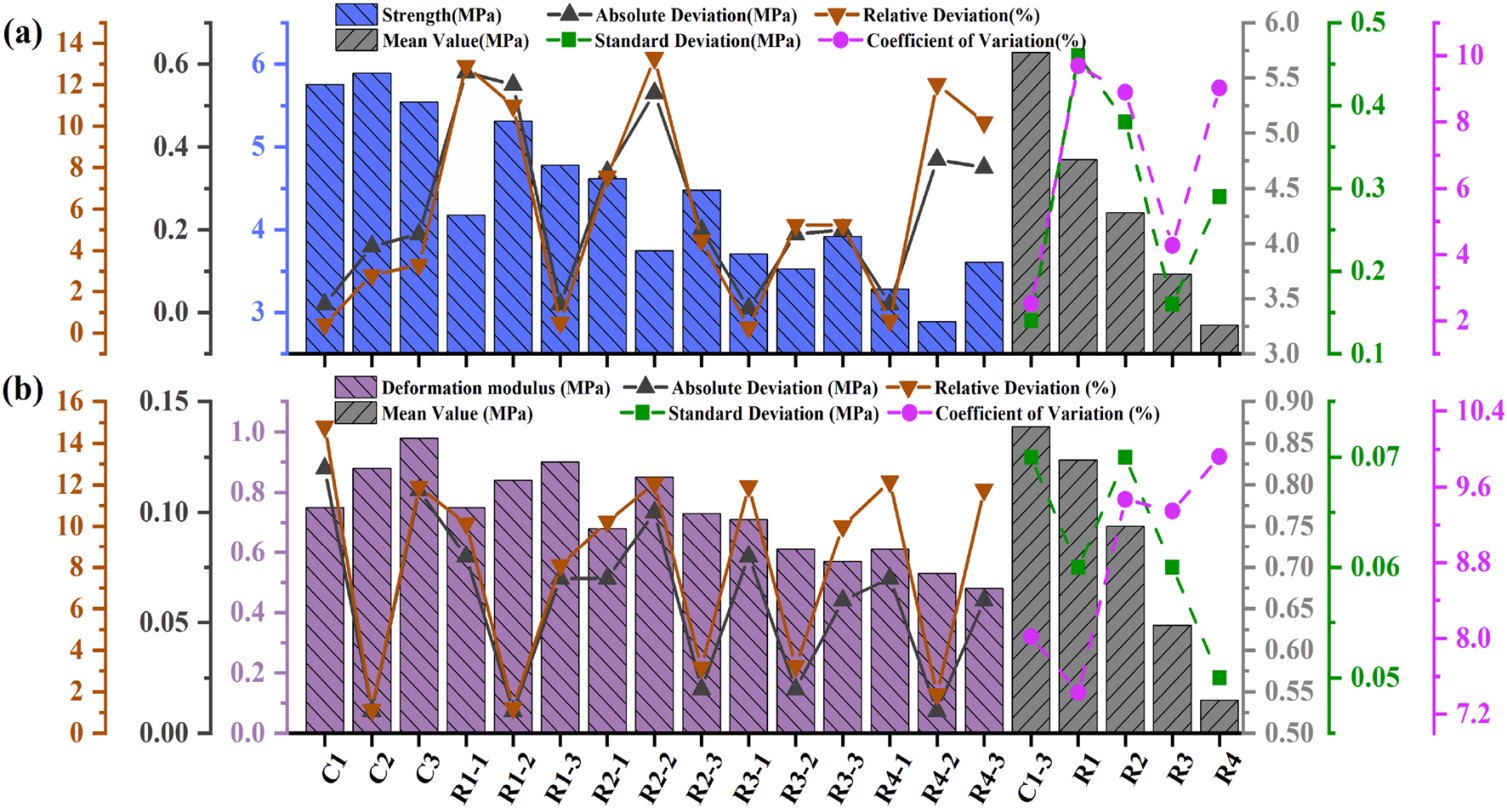
Test data processing diagram for DDRs: (**a**) The peak failure strength ; (**b**) The deformation modulus (2).

#### DDH

**Figure 8:**
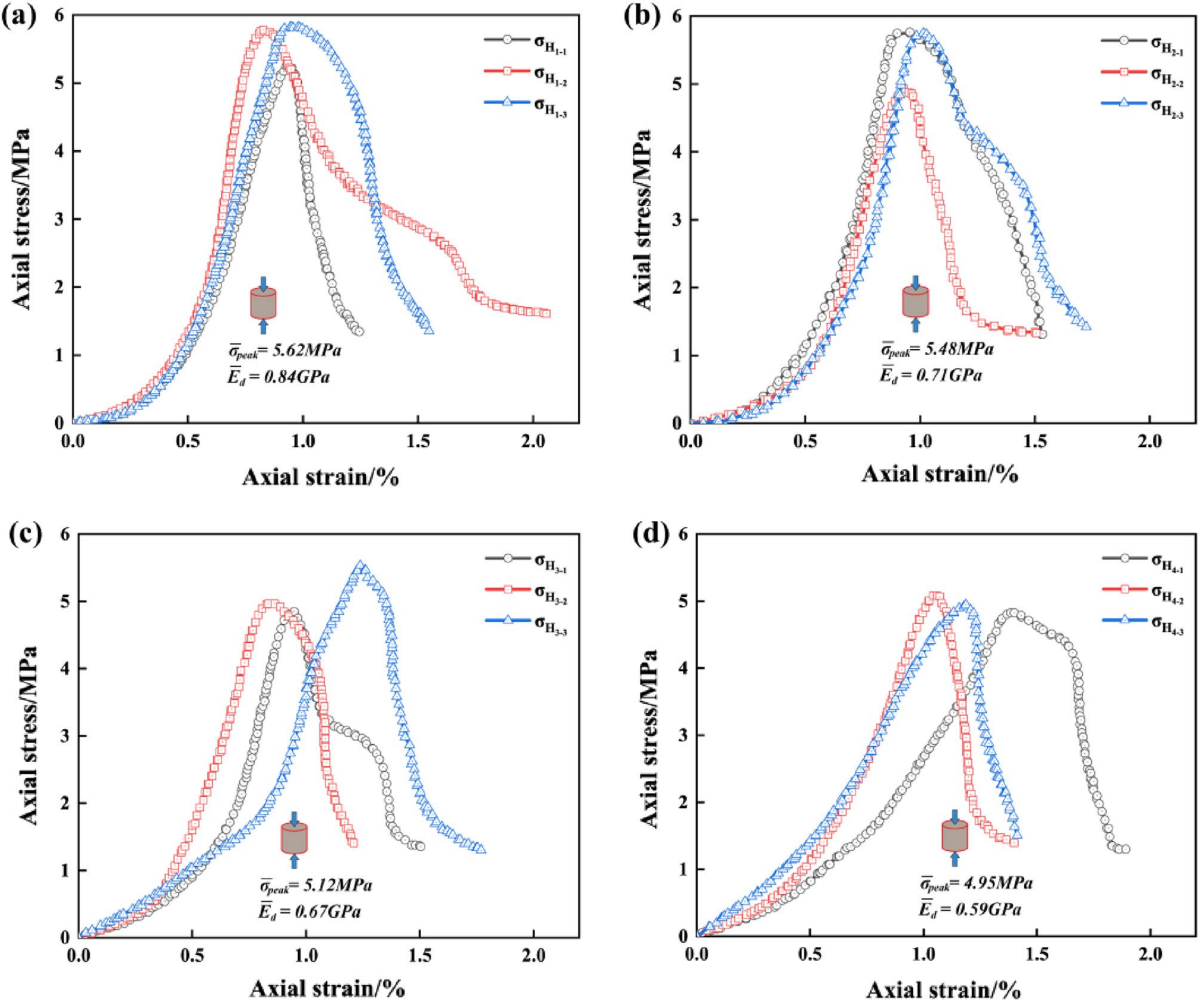
Stress–strain curves of the drilled specimens with different DDRs: (**a**) DDR =  20 mm; (**b**) DDH =  40 mm; (**c**) DDH =  60 mm; (**d**) DDH = 80 mm.

**Figure 9:**
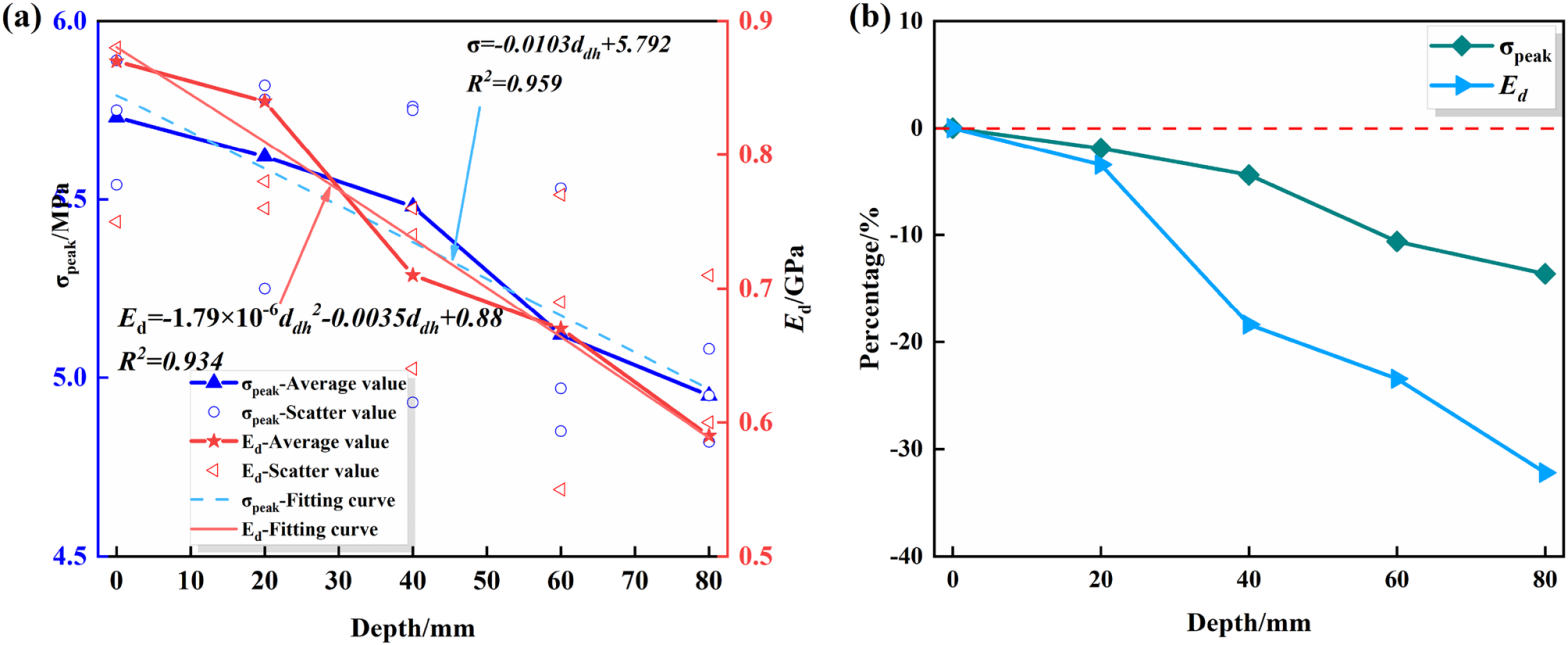
Variations of the peak strengths and deformation moduli of the drilled specimens with different DDHs: (**a**) Data and fitting curve; (**b**) Mean change rate.

1. Tables 6 and 7 indicate the mean values of the peak-failure strengths of the drilled specimens were 5.62 MPa, 5.48 MPa, 5.12 MPa, and 4.95 MPa and the mean values of the deformation moduli are 0.84 GPa, 0.71 GPa, 0.67 GPa, 0.59 GPa when the DDHs were 20 mm, 40 mm, 60 mm, and 80 mm, respectively. The peak-failure strength of the specimens is negatively correlated with the DDH, indicating that a deeper DDH leads to a lower bearing capacity. As can be seen from Figs. 8 and 9, the mean values of the peak-failure strength of the four different borehole depths decrease by 1.9%, 4.4%, 10.6%, and 13.6%, and the mean values of the deformation modulus of the four different borehole diameters decrease by 3.45%, 18.39%, 23.45%, and 32.18%, respectively, compared to the intact specimens.
2. With the increasing drilling depth, the peak-failure strength and the deformation modulus of the specimen decreases, and overall both of them are negatively correlated with the drilling depth. The fit equations are σ = − 0.0103d_dh_ + 5.792, and E = − 1.79 × 10^-6^d_dh_^2^ − 0.0035d_dh_ + 0.88. The values of the goodness of fit are 0.959 and 0.934, and the goodness of fit is fine.
3. In a comprehensive view, as the drilling depth and the unloading range continues to increase, the stress–strain curve of the specimen is more likely to enter the yielding stage during the loading process, and the weakening effect on the mechanical properties of the rock is more significant.

**Figure 10:**
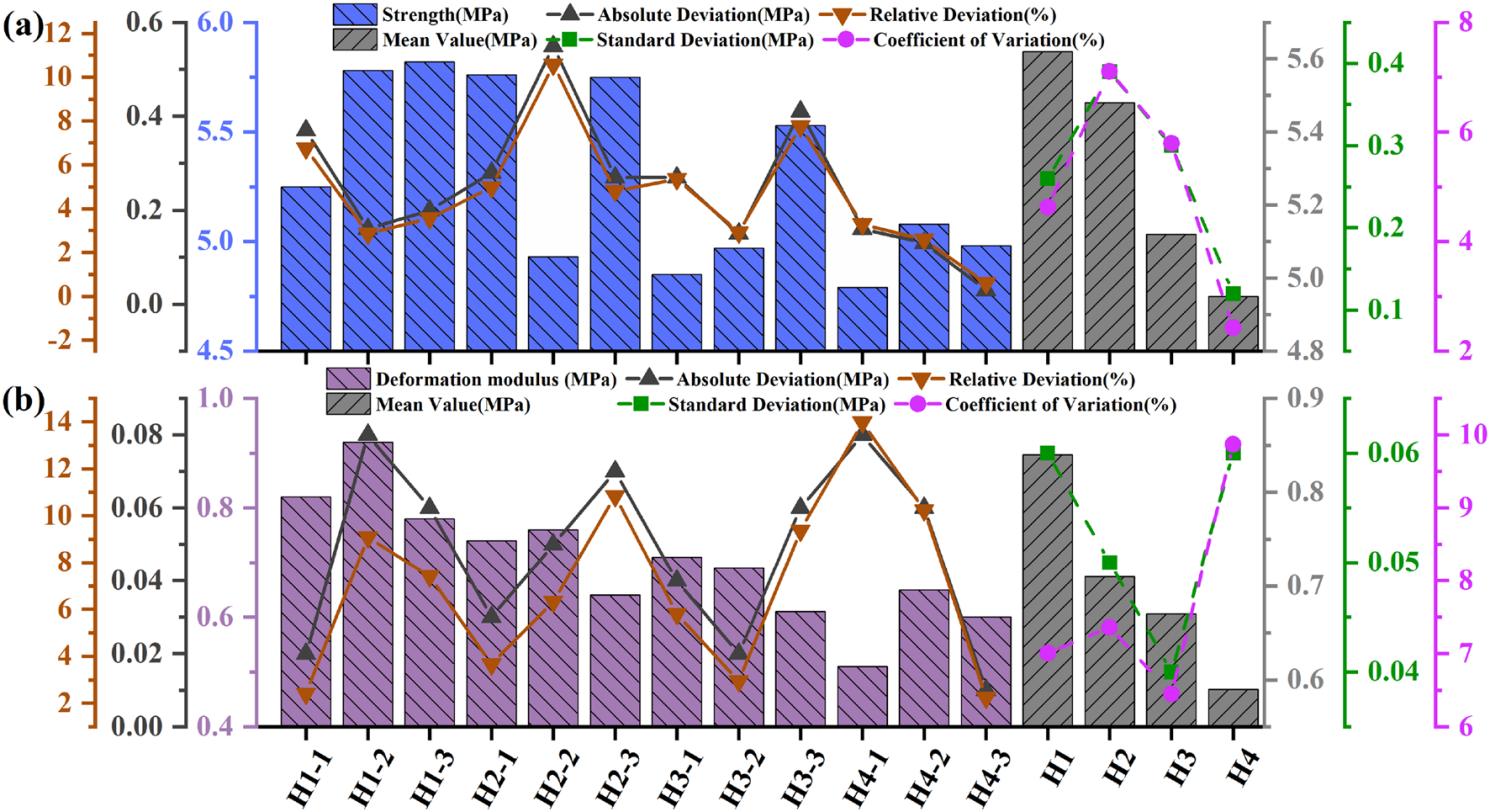
Test data processing diagram for DDHs: (**a**) The peak failure strength; (**b**) The deformation modulus.

#### DS

**Figure 11:**
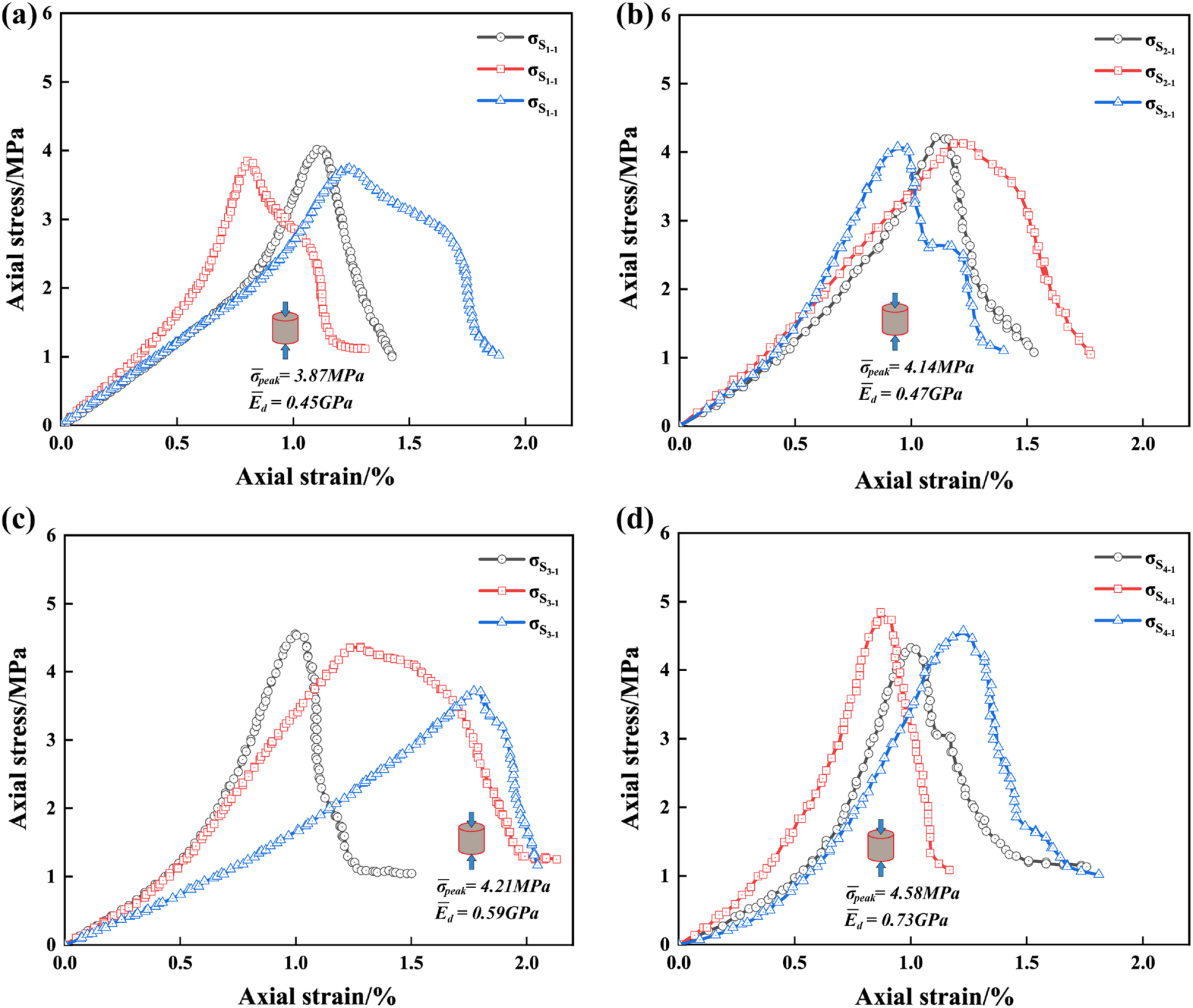
Stress–strain curves of the drilled specimens with different DSs: (**a**) DS = 30 mm; (**b**) DS = 35 mm; (**c**) DS = 40 mm; (**d**) DS = 45 mm.

**Figure 12:**
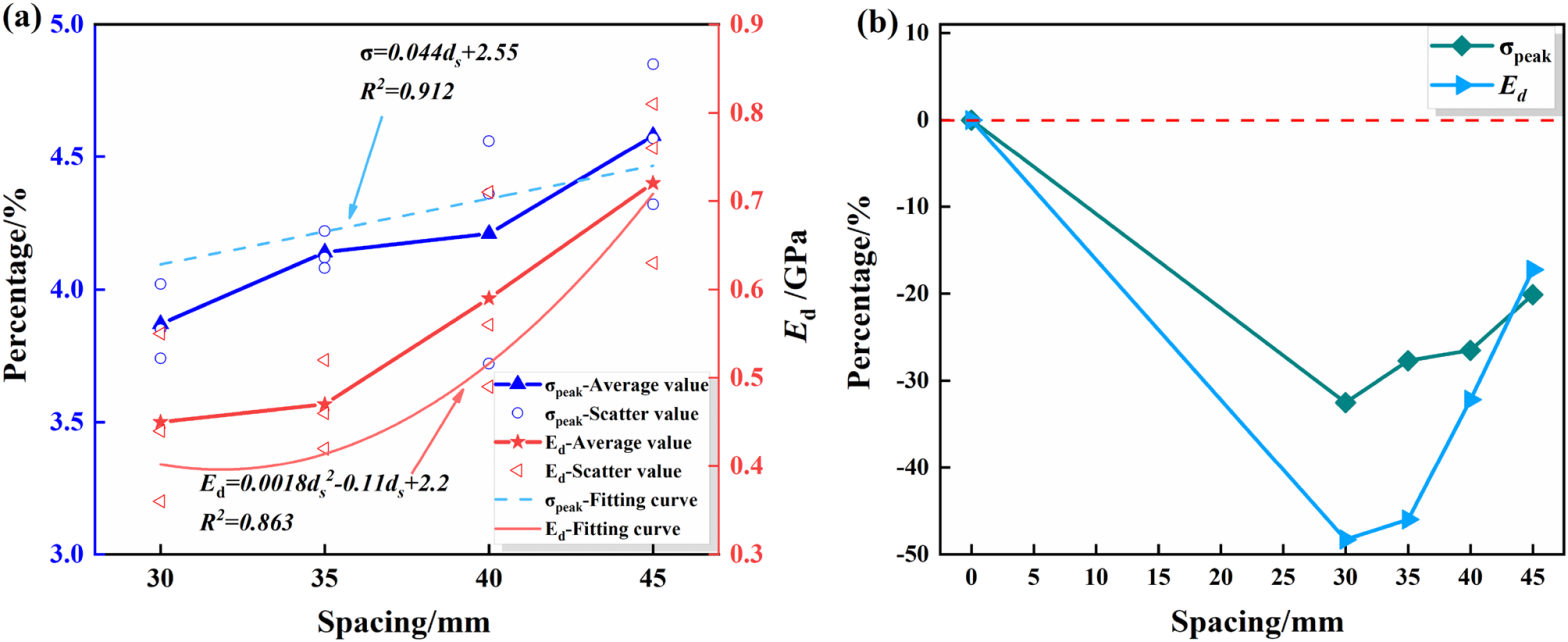
Variations of the peak strength and deformation modulus of drilling specimen with different DSs: (**a**) Data and fitting curve; (**b**) Mean change rate.

1. Tables 8 and 9 indicate the mean values of the peak strengths of the drilled specimens are 3.87 MPa, 4.14 MPa, 4.21 MPa, and 4.58 MPa, and the mean values of the deformation moduli are 0.45 GPa, 0.47 GPa, 0.59 GPa, 0.73 GPa, for DS of 30 mm, 35 mm, 40 mm, and 45 mm, respectively. The peak-failure strengths of the specimens are positively correlated with the DS. As can be seen from Figs. 11 and 12, the average values of the peak-failure strength of drilled specimens with different hole spacing decrease by 32.5%, 27.7%, 26.5%, and 20.1%, and the mean values of the deformation moduli of the four different borehole diameters decrease by 48.28%, 45.98%, 32.18%, and 17.24%, respectively, compared to the intact specimens.
2. With the increasing drilling spacing, the peak-failure strength of the specimen increases and then decreases, and the deformation modulus continues to grow, but overall both of them are positively correlated with the drilling spacing. The fitting functions are σ = 0.044d_s_ + 2.55, and E = 0.0018d_s_^2^ − 0.11d_s_ + 2.2. The values of the goodness of fit are 0.912 and 0.863, respectively, and the goodness of fit is fine. When the hole distance exceeds 35 mm, the growth rate of the deformation modulus of the drilled specimen is faster, which indicates that the stress state of the specimen is changed and the overall strength is increased.
3. With the increase of drilling spacing, the stress value required for the specimen to enter the yielding stage continues to increase, indicating that the smaller spacing has a better effect on the weakening of rock mechanical properties, and the stress fields in the drilling holes affect each other, forming a larger unloading area.

**Figure 13:**
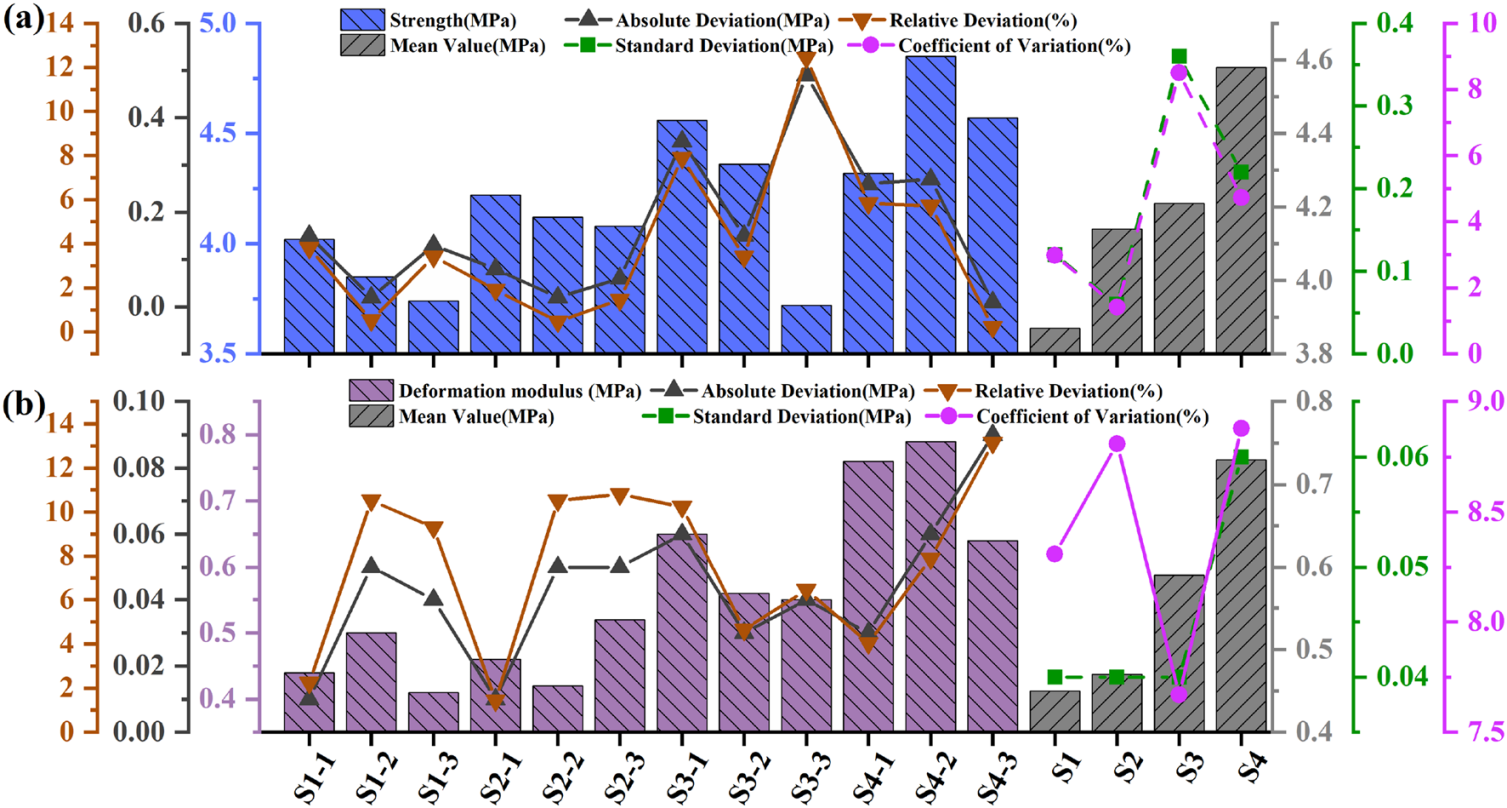
Test data processing diagram for DSs: (**a**) The peak failure strength ; (**b**) The deformation modulus

### Significance analysis

The above test data were extracted for 36 sets of peak-failure strength and deformation modulus data of drilled specimens, with the intact specimens used as the control group. The correlation between each influencing factor and the peak-failure strength and deformation modulus of drilled specimens was quantitatively calculated using Grey Relational Analysis. The significance of each influence on the peak-failure strength and deformation modulus of drilled specimens was determined, as listed in Table 10.

**Table 10:** Data on factors influencing the peak-failure strength and deformation modulus of drilled specimens.

Number	Specimen number	The peak-failure strength/MPa	The deformation modulus/GPa	DDR/mm	DDH/mm	DS/mm
1	R_1-1_	4.18	0.75	10	100	0
2	R_1-2_	5.31	0.84	10	100	0
3	R_1-3_	4.78	0.9	10	100	0
4	R_2-1_	4.62	0.68	15	100	0
5	R_2-2_	3.75	0.85	15	100	0
6	R_2-3_	4.48	0.73	15	100	0
7	R_3-1_	3.71	0.71	20	100	0
8	R_3-2_	3.53	0.61	20	100	0
9	R_3-3_	3.92	0.57	20	100	0
10	R_4-1_	3.28	0.61	25	100	0
11	R_4-2_	2.89	0.53	25	100	0
12	R_4-3_	3.61	0.48	25	100	0
13	H_1-1_	5.25	0.82	10	20	0
14	H_1-2_	5.78	0.92	10	20	0
15	H_1-3_	5.82	0.78	10	20	0
16	H_2-1_	5.76	0.74	10	40	0
17	H_2-2_	4.93	0.76	10	40	0
18	H_2-3_	5.75	0.64	10	40	0
19	H_3-1_	4.85	0.71	10	60	0
20	H_3-2_	4.97	0.69	10	60	0
21	H_3-3_	5.53	0.61	10	60	0
22	H_4-1_	4.79	0.51	10	80	0
23	H_4-2_	5.08	0.65	10	80	0
24	H_4-3_	4.98	0.6	10	80	0
25	S_1-1_	4.02	0.44	10	100	30
26	S_1-2_	3.85	0.5	10	100	30
27	S_1-3_	3.74	0.41	10	100	30
28	S_2-1_	4.22	0.46	10	100	35
29	S_2-2_	4.12	0.42	10	100	35
30	S_2-3_	4.08	0.52	10	100	35
31	S_3-1_	4.56	0.65	10	100	40
32	S_3-2_	4.36	0.56	10	100	40
33	S_3-3_	3.72	0.55	10	100	40
34	S_4-1_	4.32	0.76	10	100	45
35	S_4-2_	4.85	0.79	10	100	45
36	S_4-3_	4.57	0.64	10	100	45

#### Analysis series determination

The peak-failure strength and deformation modulus of the drilled specimen were used as the reference sequences, and the drill hole diameter, depth, and spacing were used as the comparison sequences. The analysis sequence can be expressed as follows:

$$\omega_{i} \left( k \right) = \left( {\omega_{i} \left( 1 \right),\omega_{i} \left( 2 \right),...,\omega_{i} \left( {36} \right)} \right)$$, where *k* = 1, 2, … 36; *i* = 0, 1, 2, 3, 4.

#### Dimensionless variables

The sequences were homogenized, and the formulae were calculated as shown below:

$$x_{i} \left( k \right) = AVG\omega_{i} \left( k \right)$$, *k* = 1, 2, …, 36; *i* = *0, 1, 2, 3, 4*.

#### Calculate the correlation degree

The absolute difference series of the comparison sequence and the reference sequence were found separately, and the two levels of the maximum difference and two levels of the minimum difference were found. The calculation is shown below:

1 $$\Delta_{0i} \left( k \right) = \left| {x_{0} \left( k \right) - x_{i} \left( k \right)} \right|,\;k = {1},{2}, \ldots ,{36};i = 0,1,2,3,4.$$

The maximum difference and minimum difference of the sequence are:

$$\Delta_{{0i\sigma - {\text{peak}}}} \left( {\max } \right) = \mathop {\max }\limits_{i} \mathop {\max }\limits_{k} \Delta_{0i} \left( k \right) = 2.639763;\;\;\Delta_{0iE} \left( {\max } \right) = \mathop {\max }\limits_{i} \mathop {\max }\limits_{k} \Delta_{0i} \left( k \right) = 1.329453.$$

$$\Delta_{{0i\sigma - {\text{peak}}}} \left( {\min } \right) = \mathop {\min }\limits_{i} \mathop {\min }\limits_{k} \Delta_{0i} \left( k \right) = 0.019709;\;\; \Delta_{0iE} \left( {\min } \right) = \mathop {\min }\limits_{i} \mathop {\min }\limits_{k} \Delta_{0i} \left( k \right) = 0.012573.$$

The formula for calculating the correlation coefficient between $x_{0} \left( k \right)$ and $x_{i} \left( k \right)$ is:

2 $$\varepsilon \left( {x_{0} \left( k \right),x_{i} \left( k \right)} \right) = \frac{{\mathop {\min }\limits_{i} \mathop {\min }\limits_{k} \Delta_{0i} \left( k \right) + \zeta \mathop {\max }\limits_{i} \mathop {\max }\limits_{k} \Delta_{0i} \left( k \right)}}{{\Delta_{0i} \left( k \right) + \zeta \mathop {\max }\limits_{i} \mathop {\max }\limits_{k} \Delta_{0i} \left( k \right)}}$$

where $\zeta$ is usually taken as = 0.5, and the average value of the calculated correlation coefficient is taken as the correlation between the reference sequence and the comparison sequence, calculated as follows:

3 $$\gamma \left( {x_{0} ,x_{i} } \right) = \frac{1}{36}\sum\limits_{k = 1}^{36} {\varepsilon \left( {x_{0} \left( k \right),x_{i} \left( k \right)} \right)}$$

The peak-failure strength grey correlation degrees are: $\gamma \left( {x_{0} ,x_{1} } \right)$ = 0.810846; $\gamma \left( {x_{0} ,x_{2} } \right)$ = 0.801637; $\gamma \left( {x_{0} ,x_{3} } \right)$ = 0.515757.

The deformation modulus grey correlation degrees are: $\gamma \left( {x_{0} ,x_{1} } \right)$ = 0.745433; $\gamma \left( {x_{0} ,x_{2} } \right)$ = 0.688428; $\gamma \left( {x_{0} ,x_{3} } \right)$ = 0.437129.

The calculated correlations are listed in Table 11, and the correlations causing changes in the peak-failure strength and deformation modulus of the drilled specimens are ranked the drilling diameter (DDR) being the highest, followed by DDH and DS.

**Table 11:** Ranking of the correlations of the influencing factors.

Influencing factor	The peak-failure strength		The deformation modulus	
	Grey correlation degree	Sort by association	Grey correlation degree	Sort by association
DDR	0.810846	1	0.745433	1
DDH	0.801637	2	0.688428	2
DS	0.515757	3	0.437129	3

The DDR has the most significant impact on the peak-failure strength and deformation modulus of the specimens, followed by the depth and spacing. In practical engineering applications, the diameter and depth of drilling holes can be adjusted according to the basic situation of the site to achieve a better pressure relief effect.

### Effects of the drilling parameters on damage characteristics of specimens

#### DDR

**Figure 14:**
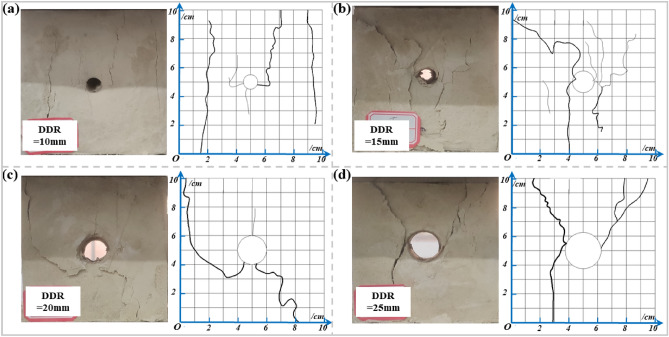
Damage pattern of the specimens from different DDRs: (**a**) DDR = 10 mm. (**b**) DDR = 15 mm; (**c**) DDR = 20 mm; (**d**) DDR = 25 mm.

1. As seen from Fig. 14, damage to the specimen is mainly spalling, crack expansion, and specimen skin drop damage in the inner wall of the borehole. All specimens with different borehole diameters show tensile cracks due to the stress concentration around the borehole, and transverse cracks are formed from the borehole outward with the increasing load in the vertical direction^49–51^. Figure 14a shows that the connection between the initiating crack and the borehole is weak for a borehole diameter of 10 mm. Figure 14b shows that at a borehole diameter of 15 mm, the initiating crack is produced near the borehole, and inner wall spalling occurs. Multiple cracks connected to the borehole extend deeper into the specimen, but the crack width is small, and small tensile cracks are produced around the borehole in addition to the main crack. When the diameter of the borehole is 20 mm, debris flaking and the falling phenomenon are more prominent, the crack is more clearly defined, and the crack width increases (Fig. 14c). Figure 14d shows that the cracks were produced earlier, and the main crack width continued to grow at a borehole diameter of 25 mm.
2. During the compression of the specimen, it is mainly affected by the main stress field and the borehole stress field, and with the increase of vertical load, the borehole stress field is superimposed on the main stress field, which mainly shows that the damage occurs in the borehole structure and cracks in the direction perpendicular to the maximum main stress (horizontal direction), and subsequently expands along the direction of the maximum main stress.
3. Overall, as the diameter of the borehole increases, the tiny cracks gradually disappear and evolve into a smaller number of cracks with larger widths and lengths. When the DDR exceeds 15 mm, the damage pattern of the specimen changes, and fissures start to develop away from the borehole extension. It indicates that the specimens are mainly influenced by the main stress field and the influence of the borehole stress field is gradually weakened.

#### DDH

**Figure 15:**
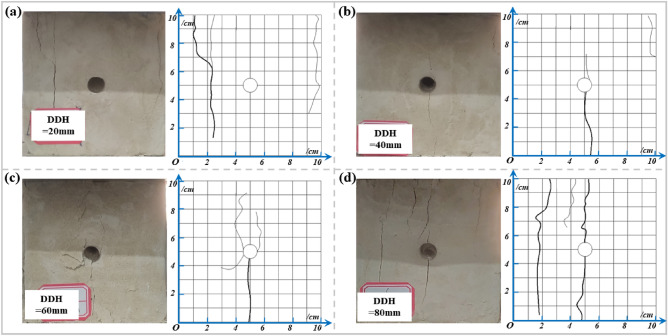
Damage pattern of the specimens from different DDHs: (**a**) DDH = 20 mm; (**b**) DDH = 40 mm; (**c**) DDH = 60 mm; (**d**) DDH = 80 mm.

1. As seen in Fig. 15, the main damage form of the specimen is still crack expansion and epidermal drop. Among them, the specimens with greater hole depths have tensile cracks due to stress concentration around the borehole. With the increasing load in the vertical direction, macro cracks are formed through the borehole outward, while the cracks in the borehole with smaller hole depth mainly appear in the distant part of the borehole. Figure 15a shows that the connection between the starting crack and the borehole is not close when the borehole depth is 20 mm, and the cracks mainly develop at the distal part of the borehole. Figure 15b shows that initiation cracks form around the borehole at a hole depth of 40 mm, but the cracks are relatively inconspicuous and small in width. Tensile cracks are also produced far from the borehole area. Figure 15c shows that at a hole depth of 60 mm, in addition to the main crack, small tensile cracks form around the hole, and the cracks gradually expand deeper. Figure 15d shows that at a hole depth of 80 mm, there is a main crack through the hole and the specimen, and the width of the crack is larger.
2. When the borehole depth is small, the borehole morphology is relatively intact, and the damage of the specimen occurs mainly on the sides away from the borehole. As DDH increases (more than 40 mm), the cracks keep approaching the borehole, and longitudinal cracks begin to appear near the borehole and gradually penetrate. It indicates that the influence of the borehole stress field gradually weakened and the main stress field began to dominate.

#### DS

**Figure 16:**
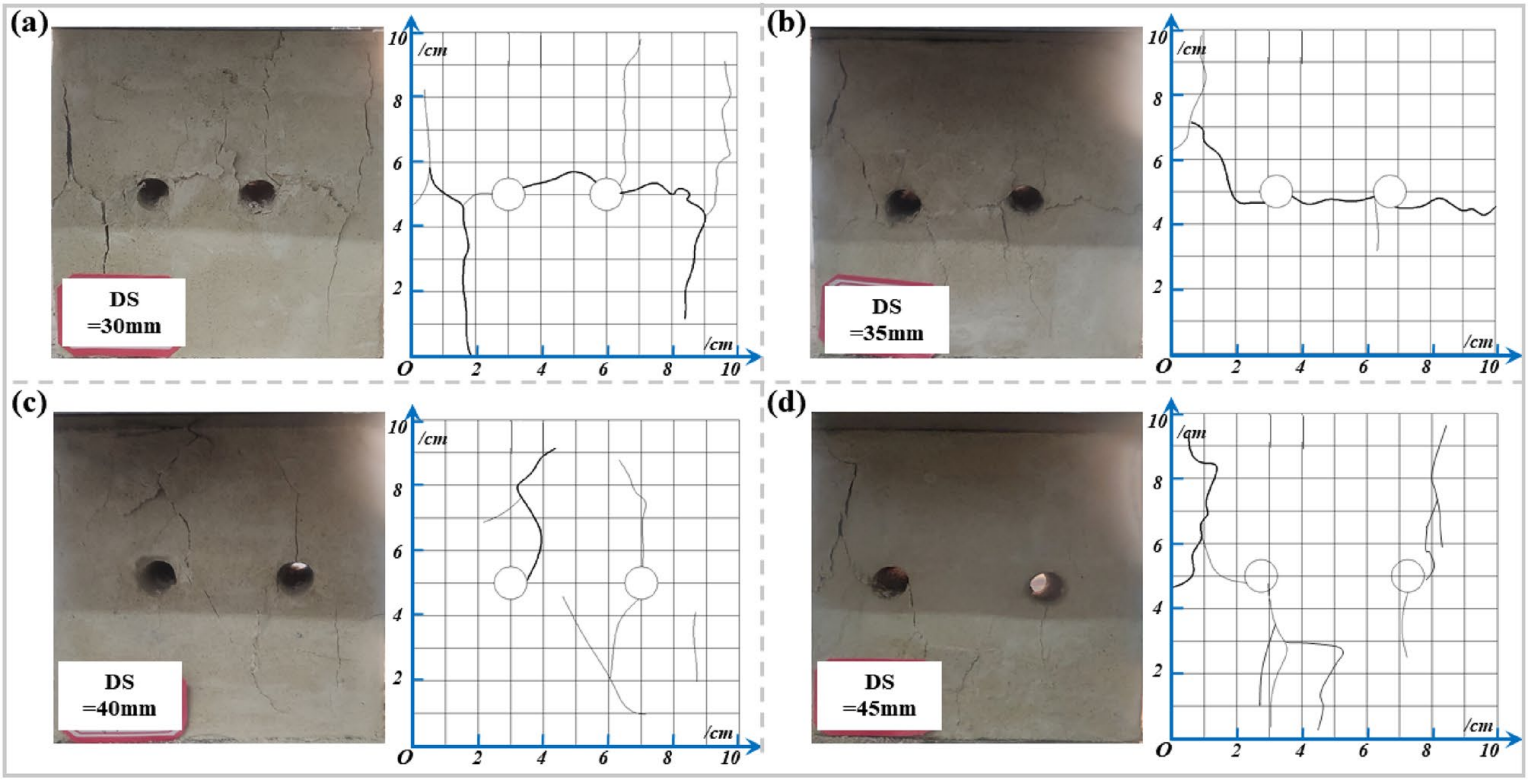
Damage pattern of the drilled specimens with different DSs: (**a**) DS = 30 mm; (**b**) DS = 35 mm; (**c**) DS = 40 mm; (**d**) DS = 45 mm.

1. As seen in Fig. 16, the primary damage forms of the specimens remain crack expansion and skin drop. The specimen with smaller spacing produces cracks around the borehole and between the two boreholes. With the increase of vertical load, cracks start along the direction perpendicular to the maximum principal stress (horizontal direction), and the cracks gradually expand and form transverse cracks through the two holes, indicating that the stress fields of the two boreholes are superimposed at this time. In contrast, the two-hole specimen with a larger spacing produces cracks only around the borehole and gradually expands to form macroscopic cracks that do not interfere. Figure 16a shows that when the DS is 30 mm, a through macro crack is produced between the two boreholes, and the crack width is greater compared to other spacing, resulting in overall structural damage of the specimen, called "through-type" damage.
2. Fig. 16b shows that at a spacing of 35 mm, a through macroscopic crack is produced between the two boreholes. However, the crack width is smaller than 30 mm. Figure 16c shows that at a spacing of 40 mm, cracks are produced between the two boreholes, but there is no penetration. As the load increases, the specimen is damaged, called "independent-penetration transition type" damage. Figure 16d shows that when the distance between the boreholes is 45 mm, the two boreholes are independent, and the interaction between the two holes is small. No through cracks are produced between the boreholes, and the cracks around the respective boreholes are far apart. As the load increases, structural damage to the specimen is caused, called "independent" damage. It also indicates that the influence range of the borehole stress field is about 1.5–2 times the borehole diameter.
3. Comparing the damage patterns of specimens with different hole spacings, as the hole spacing increases, the independence of the two boreholes continues to increase, and the mutual influence of each borehole continues to decrease. The transverse crack between the two boreholes continues to decrease until it disappears. An "independent-penetration transition" damage forms in the 40 mm borehole spacing specimens. At this time, the pressure relief of the large diameter borehole has the least influence on the anchorage area of the surrounding rock, which can achieve the organic unification of the pressure relief effect and anchorage strength. The longitudinal cracks on both sides of the specimen continue to increase, indicating that the main stress field dominates at this time.

**Figure 17:**
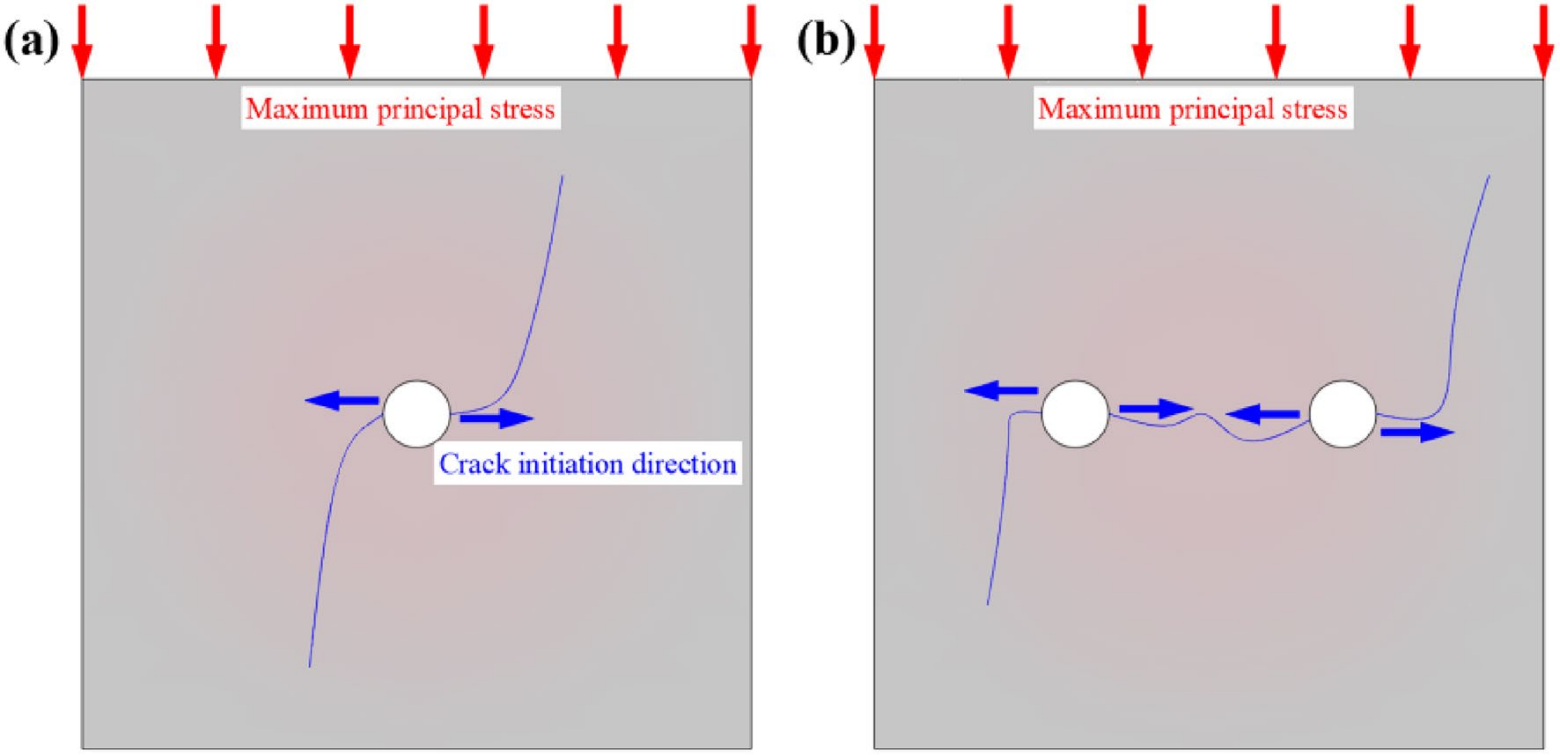
Schematic diagram of crack extension direction and maximum principal stress direction: (**a**) Single drill hole; (**b**) Multiple holes.

The vertical load provided by the testing machine was defined as the maximum principal stress in a vertical downward direction. As shown in Fig. 17a, when the depth of the borehole is large enough (100 mm), the crack initially starts in the horizontal direction of the borehole (perpendicular to the direction of the maximum principal stress), and the length of the horizontal crack is small, and the final morphology of the crack is consistent with the direction of the maximum principal stress. As shown in Fig. 17b, the crack initiation direction is still perpendicular to the direction of the maximum principal stress, and the cracks can be connected with each other when the spacing of the boreholes is small, and the horizontal cracks are longer, and the final morphology of the cracks is also consistent with the direction of the maximum principal stress. It can be seen that the initial crack expansion is more adequate when the stress fields of the boreholes are superimposed on each other.

The mechanism of pressure relief works to transfer the high stress in the shallow part of the roadway to the deep part, reduces the peak-failure strength of the surrounding rock in the drilling section, and changes the deformation modulus. After drilling, cracks are generated around the borehole, the plastic zone of the surrounding rock of the borehole is closed, the plastic energy dissipation effect is enhanced, and the deep impact conduction path is weakened to protect the roadway.

## Conclusions

1. Using the Grey Relational Analysis, the factors affecting the peak-failure strength and deformation modulus of drilled specimens were ranked in order of significance. The factors affecting the peak-failure strength and deformation modulus of drilled specimens in descending order were drilling diameter (DDR), followed by drilling depth (DDH), and drilling spacing (DS).
2. The DDR, DDH, and DS all show primary linear functions with the peak-failure strength. The DDR and DDH of single-hole specimens negatively correlate with the peak-failure strength. When more cracks are produced around the drill holes, a more complete release of strain energy of the specimen occurs. The DS of double-hole specimens and the peak-failure strength are positively correlated. As the spacing between the boreholes continues to increase, the specimen changes from "through" damage to "independent" damage, the influence between the boreholes decreases, and the strain energy released decreases.
3. The DDR, DDH, and DS all show quadratic linear functions with the deformation modulus. The DDR and DDH of single-hole specimens negatively correlate with the deformation modulus. The DS of double-hole specimens and the deformation modulus are positively correlated.
4. The mechanism of pressure relief works to transfer the high stress in the shallow part of the roadway to the deep part, reduces the peak-failure strength and the deformation modulus of the surrounding rock in the drilling section. During the compression process of the specimen, the stress field in the borehole dominates at the beginning, and cracks start along the direction perpendicular to the maximum principal stress (horizontal direction), and the maximum principal stress field dominates at the later stage, and vertical cracks of larger width appear.

## Data Availability

All data, models, or codes that support the findings of this study are available from the corresponding author upon reasonable request.

## References

[1] Malinowska AA (2016). The impact of deep underground coal mining on earth fissure occurrence. Acta Geodynamica et Geomaterialia.

[2] Rasskazov IY, Kursakin GA, Potapchuk MI (2012). Geomechanical assessment of deep-level mining conditions in the Yuzhnoe complex ore deposit. J. Min. Sci..

[3] Sakantsev GG, Sakantsev MG, Cheskidov VI, Norri VK (2014). Improvement of deep-level mining systems based on optimization of accessing and open pit mine parameters. J. Min. Sci..

[4] Qin D, Wang X, Zhang D, Chen X (2019). Study on surrounding rock-bearing structure and associated control mechanism of deep soft rock roadway under dynamic pressure. Sustainability.

[5] Wu H, Wang X, Yu W, Wang W, Zhang Z, Peng G (2020). Analysis of influence law of burial depth on surrounding rock deformation of roadway. Adv. Civil Eng..

[6] Czarny R, Malinowski M, Chamarczuk M, Ćwiękała M, Olechowski S, Isakow Z, Sierodzki P (2021). Dispersive seismic waves in a coal seam around the roadway in the presence of excavation damaged zone. Int. J. Rock Mech. Min. Sci..

[7] Frith R, Reed G, Jones A (2020). A causation mechanism for coal bursts during roadway development based on the major horizontal stress in coal: Very specific structural geology causing a localised loss of effective coal confinement and Newton’s second law. Int. J. Min. Sci. Technol..

[8] Wu G, Chen W, Jia S, Tan X, Zheng P, Tian H, Rong C (2020). Deformation characteristics of a roadway in steeply inclined formations and its improved support. Int. J. Rock Mech. Min. Sci..

[9] Xu X, He F, Li X, He W (2021). Research on mechanism and control of asymmetric deformation of gob side coal roadway with fully mechanized caving mining. Eng. Failure Anal..

[10] Zhu Q, Li T, Du Y, Zhang H, Ran J, Li W, Zhang S (2022). Failure and stability analysis of deep soft rock roadways based on true triaxial geomechanical model tests. Eng. Failure Anal..

[11] Zhan Q, Shahani NM, Zheng X, Xue Z, He Y (2022). Instability mechanism and coupling support technology of full section strong convergence roadway with a depth of 1350 m. Eng. Failure Anal..

[12] Zhang K, Li Y, Feng L, Meng X, Zhong D, Huang L (2022). Roof deformation characteristics and experimental verification of advanced coupling support system supporting roadway. Energy Sci. Eng..

[13] Yu W, Li K, Liu Z, An B, Wang P, Wu H (2021). Mechanical characteristics and deformation control of surrounding rock in weakly cemented siltstone. Environ. Earth Sci..

[14] Sinha S, Walton G (2021). Modeling the behavior of a coal pillar rib using bonded block models with emphasis on ground-support interaction. Int. J. Rock Mech. Min. Sci..

[15] Xie S, Pan H, Zeng J, Wang E, Chen D, Zhang T, Peng X, Yang J, Chen F, Qiao S (2019). A case study on control technology of surrounding rock of a large section chamber under a 1200-m deep goaf in Xingdong coal mine, China. Eng. Fail. Anal..

[16] Yang H, Zhang N, Han C, Sun C, Song G, Sun Y, Sun K (2021). Stability control of deep coal roadway under the pressure relief effect of adjacent roadway with large deformation: A case study. Sustainability.

[17] Xu L, Lu K, Pan Y, Qin Z (2022). Study on rock burst characteristics of coal mine roadway in China. Energy Sources, Part A Recov. Util. Environ. Effects.

[18] Zhang C, Canbulat I, Hebblewhite B, Ward CR (2017). Assessing coal burst phenomena in mining and insights into directions for future research. Int. J. Coal Geol..

[19] He Y, Gao M, Xu D, Yu X (2021). Investigation of the evolution and control of fractures in surrounding rock under different pressure relief and support measures in mine roadways prone to rockburst events. Royal Soc. Open Sci..

[20] Yang, X., Hu, C., He, M., Wang, H., Zhou, Y., Liu, X., Zhen, E., Ma, X. Study on presplitting blasting the roof strata of adjacent roadway to control roadway deformation. Shock Vib., *2019* (2019).

[21] Ma T, Li F, Yang Y, Li L (2022). Study on energy evolution and crack propagation of rock mass under single hole uncoupled charge blasting. Appl. Eng. Sci..

[22] Huang B, Liu J, Zhang Q (2018). The reasonable breaking location of overhanging hard roof for directional hydraulic fracturing to control strong strata behaviors of gob-side entry. Int. J. Rock Mech. Min. Sci..

[23] Wang Q, He M, Yang J, Gao H, Jiang B, Yu H (2018). Study of a no-pillar mining technique with automatically formed gob-side entry retaining for longwall mining in coal mines. Int. J. Rock Mech. Min. Sci..

[24] Xu X, He M, Gao Y (2021). Study on mining pressure law and pressure relief control under influence of key layer. Min. Metall. Explor..

[25] Xu X, He M, Zhu C, Lin Y, Cao C (2019). A new calculation model of blasting damage degree—Based on fractal and tie rod damage theory. Eng. Fract. Mech..

[26] Zhang X, Hu J, Xue H, Mao W, Gao Y, Yang J, He M (2020). Innovative approach based on roof cutting by energy-gathering blasting for protecting roadways in coal mines. Tunn. Underground Space Technol..

[27] Lin B, Zhang J, Shen C, Zhang Q, Sun C (2012). Technology and application of pressure relief and permeability increase by jointly drilling and slotting coal. Int. J. Min. Sci. Technol..

[28] Shen C, Lin B, Meng F, Zhang Q, Zhai C (2012). Application of pressure relief and permeability increased by slotting a coal seam with a rotary type cutter working across rock layers. Int. J. Min. Sci. Technol..

[29] Wu H, Zhao G, Liang W (2020). Mechanical properties and fracture characteristics of pre-holed rocks subjected to uniaxial loading: A comparative analysis of five hole shapes. Theor. Appl. Fracture Mech..

[30] Zhai C, Xu J, Liu S, Qin L (2018). Investigation of the discharge law for drill cuttings used for coal outburst prediction based on different borehole diameters under various side stresses. Powder Technol..

[31] Islam MR, Shinjo R (2009). Numerical simulation of stress distributions and displacements around an entry roadway with igneous intrusion and potential sources of seam gas emission of the Barapukuria coal mine, NW Bangladesh. Int. J. Coal Geol..

[32] Lin Q, Wang S, Wan B, Lu Y, Wang Y (2020). Characterization of fracture process in sandstone: A linear correspondence between acoustic emission energy density and opening displacement gradient. Rock Mech. Rock Eng..

[33] Najafi M, Jalali S, Khalokakaie R (2014). Thermal–mechanical–numerical analysis of stress distribution in the vicinity of underground coal gasification (UCG) panels. Int. J. Coal Geol..

[34] Zhao T, Guo W, Yu F, Tan Y, Huang B, Hu S (2018). Numerical investigation of influences of drilling arrangements on the mechanical behavior and energy evolution of coal models. Adv. Civil Eng..

[35] Zhang S, Li Y, Shen B, Sun X, Gao L (2019). Effective evaluation of pressure relief drilling for reducing rock bursts and its application in underground coal mines. Int. J. Rock Mech. Min. Sci..

[36] Zhao X, Zhang H, Zhu W (2014). Fracture evolution around pre-existing cylindrical cavities in brittle rocks under uniaxial compression. Transact. Nonferrous Metals Soc. China.

[37] Yao J, Yin Y, Zhao T, Ren W, Qiu Y, Guo W (2020). Segmented enlarged diameter borehole destressing mechanism and its influence on anchorage support system. Energy Sci. Eng..

[38] Cui F, Zhang S, Chen J, Jia C (2022). Numerical study on the pressure relief characteristics of a large-diameter borehole. Appl. Sci..

[39] Zhang Y, Li Y, Zhong J, Sun L, Meng T (2023). Optimum process parameters of IN718 alloy fabricated by plasma arc additive manufacturing using Taguchi-based grey relational analysis. Mater. Today Commun..

[40] Xie B, Peng Q, Jiaqiang E, Tu Y, Wei J, Tang S, Song Y, Fu G (2022). Effects of CO addition and multi-factors optimization on hydrogen/air combustion characteristics and thermal performance based on grey relational analysis. Energy.

[41] Jiang X, Wu C, Zhou H, Gao B, Fang X, Han J, Gao W (2022). Relationship between thermal properties and structure, composition of briquette through grey relational analysis. J. Appl. Geophys..

[42] Ma N, Li J, Zhao Z (2015). Distribution of the deviatoric stress field and plastic zone in circular roadway surrounding rock. J. China Univ. Min. Technol..

[43] Yuan Z, Zhao J, Li S, Jiang Z, Huang F (2022). A unified solution for surrounding rock of roadway considering seepage, dilatancy, strain-softening and intermediate principal stress. Sustainability.

[44] Kahraman S (2001). Evaluation of simple methods for assessing the uniaxial compressive strength of rock. Int. J. Rock Mech. Min. Sci..

[45] Huang B, Guo W, Fu Z (2018). Experimental investigation of the influence of drilling arrangements on the mechanical behavior of rock models. Geotech. Geol. Eng..

[46] Liu J, Wu N, Si G, Zhao M (2021). Experimental study on mechanical properties and failure behaviour of the pre-cracked coal-rock combination. Bull. Eng. Geol. Environ..

[47] Ning J, Wang J, Jiang J, Hu S, Jiang L, Liu X (2018). Estimation of crack initiation and propagation thresholds of confined brittle coal specimens based on energy dissipation theory. Rock Mech. Rock Eng..

[48] Tang Y, Okubo S, Xu J, Peng S (2018). Study on the progressive failure characteristics of coal in uniaxial and triaxial compression conditions using 3D-digital image correlation. Energies.

[49] Chen W, Wan W, Zhao Y, Peng W (2020). Experimental study of the crack predominance of rock-like material containing parallel double fissures under uniaxial compression. Sustainability.

[50] Zhao Y, Zhang C, Wang Y, Lin H (2021). Shear-related roughness classification and strength model of natural rock joint based on fuzzy comprehensive evaluation. Int. J. Rock Mech. Min. Sci..

[51] Zhao Y, Zhang L, Wang W, Liu Q, Tang L, Cheng G (2020). Experimental study on shear behavior and a revised shear strength model for infilled rock joints. Int. J. Geomech..

